# Comparison of neurally adjusted ventilatory assist and pressure support ventilation during the early phase of weaning from mechanical ventilation - a randomised controlled study

**DOI:** 10.1186/2197-425X-3-S1-A410

**Published:** 2015-10-01

**Authors:** A Demoule, M Clavel, C Rolland-Debord, S Perbet, N Terzi, A Kouatchet, F Wallet, C Guerin, H Roze, F Vargas, J Dellamonica, S Jaber, T Similowski

**Affiliations:** Groupe Hospitalier Pitié-Salpêtrière, Paris, France; UMRS 1158, INSERM and Pierre and Marie Curie University, Paris, France; Hôpital Dupuytren, Limoges, France; Hôpital D'Estaing, Clermont-Ferrand, France; CHU Côte de Nacre, Caen, France; CHRU, Angers, France; Centre Hospitalier Lyon-Sud, Lyon, France; Hôpital de la Croix Rousse, Lyon, France; CHU de Bordeaux, Pessac, France; Hopital Pellegrin - CHU de Bordeaux, Bordeaux, France; Hôpital de l'Archet, Nice, France; Hôpital Saint-Eloi, Montpellier, France

## Rational

Neurally adjust ventilatory assist (NAVA) is a ventilatory mode that tailors the level of assistance delivered by the ventilator to the electromyographic activity of the diaphragm. Physiological studies have demonstrated the benefit of NAVA on patient ventilator interactions and prevention of lung over inflation. However, clinical data are currently lacking.

## Objectives

To compare NAVA and Pressure Support Ventilation (PSV) at the early phase of ventilator weaning in patients recovering from an acute respiratory failure.

## Patients and Methods

A multicenter randomized controlled trial of 128 intubated adults (median [IQR] age, 66 [57-77] years) was conducted in 11 intensive care units in France from April 2010 to June 2012. Patients were included as soon as they could tolerate PSV with a PS level ≤30 cmH2O, a PEEP ≤8 cmH2O and a FiO2 ≤50%. Patients were randomly assigned to NAVA (n = 62) or PSV (n = 66). The PS level in the PSV group and the NAVA level in the NAVA group were set to obtain a tidal volume of 6-8 ml/kg. The primary end point was the probability to remain in an assist mode during the entire first 48 hours. Secondary end points were duration of mechanical ventilation and 28 days mortality.

## Results

67.2% (n = 45) in the NAVA group vs. 63.3% (n = 44) in the PSV group (p = 0.66).. The time spend in an assist mode during the first 48 hours was 47 [43-48] hours in the NAVA group vs. 47 [40-48] hours in the PSV group (p = 0.55). The asynchrony index was lower in the NAVA group (19.7% vs. 32.6%), so was the prevalence of dyspnea at day-1 (9% vs. 19%). The time to first extubation was 7 [2-10] days in the NAVA group vs. 7 [2-10] days in the PSV group (p = 0.78). The hospital mortality rate in the NAVA group was 14.5% (n = 9) vs. 21.2% (n = 14) in the PSV group (p = 0.2). NAVA and PSV were associated to similar oxygenation. NAVA was not associated with any adverse event.

## Conclusions

NAVA can be applied efficiently in a clinical setting and improves patient ventilator interaction. However, NAVA does not increase the probability to remain in an assisted mode nor it reduces hospital mortality.

